# Evaluation of the Role of Taurine in Mitigating the Deleterious Effects of Tartrazine on the Kidneys in Rats: Experimental Study

**DOI:** 10.5146/tjpath.2025.14451

**Published:** 2026-01-31

**Authors:** Abdelmonem Awad Hegazy, Walaa Abdel Haliem Rashad, Gehad Mohammad Elsayed Ali, Mai Ahmed Gobran, Basma A. Ibrahim, Emtethal Mamdouh El-Bestawy

**Affiliations:** Department of Human Anatomy and Embryology, Zagazig University, Faculty of Medicine, Zagazig, Egypt; Department of Basic Medical Science, Zarqa University, Faculty of Dentistry, Zaraqa, Jordan; Department of Human Anatomy and Embryology, Zagazig University, Faculty of Medicine, Zagazig, Egypt; Departments of Pathology, Faculty of Medicine, Zagazig University, Zagazig, Egypt; Departments of Medical Biochemistry and Molecular Biology Department, Faculty of Medicine, Zagazig University, Zagazig, Egypt

**Keywords:** Food coloring agents, Caspase-3, Proliferating cell nuclear antigen, Electron microscopy, Oxidative stress

## Abstract

**Objective:** Tartrazine (TZ) is an anionic azo dye widely used to color food products, pharmaceuticals, and cosmetics; however, its harmful effects on the kidneys are still unclear and need to be confirmed. Meanwhile, taurine (TA) is a natural antioxidant amino acid that can provide protection against various forms of glomerulonephritis. Our aim was to study the structural, and biochemical effects of TZ on the kidney and evaluate the potential protection provided by taurine.

**Material and Methods:** We used 28 adult male albino rats equally divided into 4 groups. The control group did not receive TZ or TA. The TA group received 100 mg/kg/day of TA. The TZ group received 100 mg/kg/day of TZ dissolved in distilled water. The TZ/TA group received both TZ and TA. Animal blood samples were obtained to estimate blood urea, creatinine, and random glucose levels. Kidneys samples were examined for structure as well as oxidative enzymes and kidney injury molecule 1 (KIM-1).

**Results:** Compared to the control group, the TZ group showed hyperglycemia, increased markers of oxidative stress, and shrunken, lobulated glomeruli with mesangial expansion, pyknosis, and vacuolation in the tubular lining. There was also strong immunoreactivity for PCNA and caspase-3, a thickened glomerular capillary basement membrane lacking fenestrations, swollen mitochondria with destructed cristae, and increased expression of the KIM-1. In the TZ/TA group, the convoluted tubules mostly retained the normal histological structure, but some tubules still showed a wide lumen and nuclear pyknosis of lining cells. Oxidative markers and random blood glucose levels were significantly reduced.

**Conclusion:** TZ is suggested to cause adverse kidney effects in rats, including kidney injury and structural changes, which can be mitigated by co-administration with TA.

## INTRODUCTION

Food safety is a human concern, especially with the widespread use of food and pharmaceutical dyes. Although the use of these dyes is approved by the U.S. Food and Drug Administration and the World Health Organization, their misuse, especially in quantities exceeding the permitted limits, can pose a significant risk to public health. One of these commonly used dyes is tartrazine (TZ) (1).

TZ is a synthetic, water-soluble, orange-yellow azo dye widely used to provide color in food products (i.e., soft drinks, jams, jellies, ice cream and sweets), pharmaceuticals, and cosmetics (2). Furthermore, it is used in cooking by people in some low-income countries as a low-cost alternative to saffron (3). The acceptable daily dose of TZ for humans is 7.5 to 10 mg/kg of body weight. However, there are some illegal uses that exceed the levels permitted in food. TZ consumption and its consequent harmful effects may affect population groups such as children, possibly because of their attraction to colorful foods (4).

TZ is poorly absorbed, usually less than 5%, in the intestine and therefore the small amounts absorbed are not metabolized and are excreted unchanged in the urine by the kidneys. The large unabsorbed portion of TZ is subjected to reductive metabolism by gut microbiota. Small amounts of these metabolites, such as sulfanilic acid and aniline derivatives, are also absorbed by the intestinal mucosa and excreted in the urine (5). These metabolites of TZ can be absorbed by the gastrointestinal mucosa to a greater extent than TZ itself (4). Sulfanilic acid has been reported to induce oxidative stress, cellular damage and brain toxicity (6).

In addition, TZ has been reported to induce histopathological changes and functional disturbance in various organs including the liver and kidney. The functional disturbance has been demonstrated by increased serum urea, creatinine, aspartate aminotransferase, and alanine aminotransferase (7). TZ can adversely affect the histological structure of both the endocrine and exocrine parts of the pancreas. It induces vacuolation and necrosis in the pancreatic acini, and cellular degeneration in the islets of Langerhans (8). The reported alteration in endocrine and exocrine pancreatic functions associated with TZ results in disturbance of pancreatic enzyme activities and glucose homeostasis (9). Despite the harmful effects of TZ on fish, its toxicity remains unclear and needs to be confirmed (1).

Taurine (TA), 2-aminoethanesulfonic acid, is a naturally occurring intracellular amino acid found in many animal tissues including the kidney, liver, heart, retina, and brain (10). It is a non-essential amino acid that is not involved in protein synthesis or gluconeogenesis and therefore does not provide a direct source of energy. TA is a cell-protective molecule. Due to its antioxidant nature, TA appears as a promising therapeutic approach against disorders and defects in many body systems and biological processes associated with the central nervous system, cardiovascular system, skeletal muscles, and metabolism (11). TA is a beneficial and safe agent for maintaining liver function, and it also prevents elevated blood ammonia levels as a harmful consequence of liver injury (12). In addition, it can provide protection against various forms of glomerulonephritis and retinal degeneration (13,14). TA also mitigates the effects of oxidative stress in the retina, restores the oxidant/antioxidant balance, and prevents lipid peroxidation (15).

TA is involved in various physiological and biological processes in the kidneys, which are often reflected in urine excretion patterns (13). Therefore, we aimed to elucidate the structural and biochemical effects of TZ on the kidney and evaluate the potential protection provided by TA and its underlying mechanisms.

## MATERIALS AND METHODS

### Chemicals and Reagents

TZ, in powder form Purity 86.7%, was purchased from, Sigma-Aldrich chemicals. TA in powder form Kosher, ≥ 98%, was purchased from Sigma-Aldrich chemicals.

### Animals

We used 28 adult male albino Wistar rats, weighing 180 to 200 g. Rats were purchased from the scientific and medical research center (ZSMRC), Faculty of Medicine. The animals were housed in polypropylene plastic cages at room temperature between 24 and 26°C and received a well-balanced diet in the form of chow and were allowed free access to food. The rats were allowed to acclimate to the laboratory environment for one week before starting the experimental procedures.

### Study Design

The rats were divided into 4 equal groups, each containing 7 rats as follows: Control group given a balanced diet and distilled water for 30 days without the addition of any other substances or chemicals; TA group, received 100 mg/kg/day of TA orally via gavage for 30 days (16); TZ group received 100 mg/kg/day of TZ dissolved in distilled water orally via gavage for 30 days (17); and TZ/TA group received both 100 mg/kg/day of TZ and 100 mg/kg/day of TA by oral gavage for 30 days. On the 31st day of the experimental period, the rats were anesthetized with thiopental for further experimental procedures.

### Blood Collection and Biochemical Assessment

Venous blood samples were obtained from the retroorbital venous plexuses using microcapillary tubes (18). A 2 ml blood sample was incubated in a centrifuge tube at 37°C until the blood clotted and then centrifuged to separate the serum. The serum was stored at -20°C for biochemical analysis. Serum urea and creatinine levels were quantified using BioVision colorimetric assay kits (Cat. No. K375-100 for urea; Cat. No. K625-100 for creatinine), while random glucose was measured via the hexokinase method using Glucose (HK) kit (Cat. No. GAHK20) (19). All assays were performed according to manufacturers’ protocols.

### Tissue Sample Extraction

The abdomen of each anesthetized rat was dissected, and both kidneys were extracted. One kidney was prepared for light microscopy (LM), while the other was used for electron microscopy (EM), identification of oxidation markers, and molecular analysis.

### Oxidative Stress and Antioxidant Biomarkers Estimation

After surgical extraction, kidneys were perfused with heparinized (0.16 mg/mL) phosphate-buffered saline (PBS, pH 7.4) to remove blood components. The purified tissues were flash-frozen and stored at -80°C until analysis. For homogenization, 1 g of tissue was processed in 5-10 mL of ice-cold 50 mM potassium phosphate buffer (pH 7.5), followed by centrifugation at 4000 ×g for 15 min at 4°C. The supernatant was used for Malondialdehyde (MDA) quantification via thiobarbituric acid reactive substances method using BioDiagnostic kit (MD 2529) (20). Antioxidant capacity was assessed by measuring reduced glutathione (GSH) levels using the enzymatic recycling assay (GR 2511 kit) (21), catalase (CAT) activity via hydrogen peroxide decomposition (CA 2517 kit) (22), and superoxide dismutase (SOD) activity through inhibition of phenazine methosulfate-mediated nitro-blue tetrazolium reduction (SD 2521 kit) (23). All spectrophotometric analyses were performed in accordance with the manufacturers’ protocols.

### LM Assessment

Kidney samples were immersed in 10% formaldehyde solution for 2 days and processed for LM examination (24). Sections were cut at 5 μm thickness for staining with hematoxylin and eosin (H&E), Periodic acid-Schiff (PAS) stain, and Mallory trichrome (MT) (25).

### Immunohistochemical Assessment of Caspase-3 (CASP3) and Proliferating Cell Nuclear Antigen (PCNA)

Kidney sections were immunohistochemically stained using primary rabbit monoclonoal antibody to CASP3 (Lab Vision Laboratories, Cat. #: 1475-1) and primary mouse monoclonal antibody to PCNA (Clone PC10, DAKO A/S Denmark) as specified by the data sheet. The slides were incubated with the primary antibody overnight at +4°C. Observation of the primary antibody binding was done using avidin biotin-peroxidase detection system (DAKO, Carpentaria, USA). The sections were stained with diaminobenzene as a chromogen and then counterstained with hematoxylin (26).

### Transmission EM Assessment

Kidney samples were cut into 1 mm3 pieces and fixed in cold 2.5% glutaraldehyde in 0.1 M cacodylate at 4°C for 1 day, then post-fixed in 1% osmium tetroxide for 2 h and washed in the previous buffer to remove excess fixative. Specimens were dehydrated in ascending grades of alcohol. The specimens were then cleared in propylene oxide and embedded in epoxy resin. Semithin sections (1 µm thick) were obtained and mounted in a drop of water on glass slides, and then ultrathin sections (60-70 nm thick) were obtained from selected blocks and mounted on copper grids (27).

### Morphometric Assessment

Morphometric assessments of H&E-stained sections for epithelial thickness and diameter of the cortical tubules, MT-stained sections for area percentage (%) of collagen fibers, PAS-stained sections for area % of mesangial expansion, and immuno-stained sections for optical density of PCNA immunoreaction were done using Image J (FIJI) software.

### Real Time Polymerase Chain Reaction (PCR) Detection of Kidney Injury Molecule-1 (KIM-1)

Extraction and purification of the total RNA was performed by using RNeasy Mini Kit (Qiagen, Cat No.74104) as specified in the manufacturer’s guidelines. RNA concentration and purity were verified spectrophotometrically (A260/A280 ratio >1.8). cDNA synthesis was performed using the HiSenScript™ RH(-) cDNA Synthesis Kit with 5 μL template RNA, 10 μL 2X RT Reaction Solution, 1 μL Enzyme Mix solution, and 4 μL DNase/RNase Free Water in a final volume of 20 μL under the following conditions: 45°C for 1 hour (RTase reaction), then 85°C for 10 min (RTase inactivation). The QuantiTect SYBR green PCR kit (Qiagen, Cat. No. 204141) was used to perform RT-qPCR. Each 25 µL reaction contained 12.5 µL SYBR Green Master Mix, 5 µL cDNA template, 1 µL of each primer (Table 1) (28,29), and 5.5 µL nuclease-free water. Thermal parameters and the amplification cycles were done according to the following: A primary denaturation step was done at 94°C for 15 minutes, then the following 40 cycles at 94°C for 15 seconds, and then the last 40 cycles at 60°C for 30 seconds and 72°C for 30 seconds. The cycle time values of KIM-1 were normalized with β-actin. The KIM-1 relative expression levels were calculated using the 2−ΔΔCt method (30).

**Table 1:** Primer sequence used in SYBR Green real time PCR

**Genes**	**Primers (5’ to 3’)**	**GenBank ID**	**Product Size (bp)**
**Kim-1**	F: CGGTGCCTGTGAGTAAATAGAT R: CTGGCCATGACACAAATAAGAC	NM_173149.2	418
**Rat β-actin**	F: TCCTCCTGAGCGCAAGTACTCT R: GCTCAGTAACAGTCCGCCTAGAA	NM_031144.3	153

### Statistical Analysis

Statistical analysis of the collected data was performed using analysis of variance (ANOVA) and Tukey post hoc test. A P value of < 0.05 was considered statistically significant.

## RESULTS

### Biochemical Study

**Figure 1:**
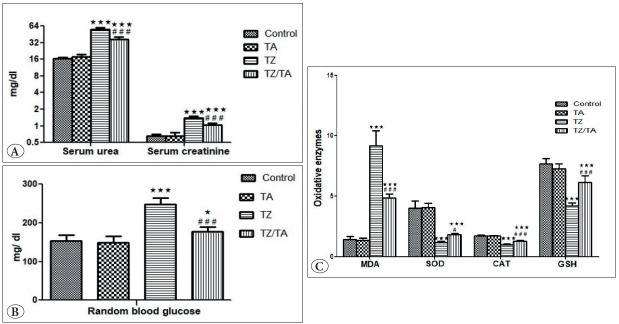
Bar charts showing statistical analysis of mean serum biomedical values in different studied groups: **A)** Urea and creatinine; **B)** Random blood glucose level; **C)** MDA, SOD, CAT and GSH. *: Comparison in relation to the control group; *: significant difference (P value < 0.05), ***: very highly significant difference (P value < 0.001). #: Comparison in relation to the TZ group; ###: very highly significant difference (P value < 0.001)

Statistical analysis of the mean values of serum urea and creatinine levels showed significantly higher levels in the TZ group compared to the control group. The TZ/TA group showed a decrease in mean serum urea and serum creatinine values, which was statistically significantly different from the TZ group and also from the control group (Table 2; Figure 1).

**Table 2:** Statistical analysis of the mean values of serum urea and creatinine, random blood glucose, MDA, SOD, CAT and GSH in the different studied groups using ANOVA and Tukey post hoc test

**Groups**	**Control (N=7)**	**TA (N=7)**	**TZ (N=7)**	**TZ + TA (N=7)**	**P**
**Parameter**	**Mean ±SD**	**Mean ±SD**	**Mean ±SD**	**Mean ±SD**	
Serum urea (mg/dl)	16.71±.81	17.87± 1.60	55.34± 3.86***	36.73 ± 3.81***,^###^	> 0.001
Serum creatinine (mg/dl)	0.65 ± 0.06	0.65 ± 0.11	1.40 ± 0.11***	1.03 ± 0.08***,^###^	> 0.001
Random blood glucose (mg/dl)	154± 14.75	149± 16.64	248 ± 17.29***	178 ± 12.13*,^###^	> 0.001
MDA (nmol/ mg)	1.42±0.28	1.35±0.20	9.17±1.26***	4.86± 0.35***,^###^	> 0.001
SOD (U/ g)	4.02±0.59	4.07±0.37	1.17±0.12***	1.82±0.14***,^#^	> 0.001
CAT (U /g)	1.74±0.04	1.71±0.05	1.01±0.05***	1.29±0.05***,^###^	> 0.001
GSH (mg/g)	7.68±0.42	7.29±0.39	4.22±0.23***	6.13±0.57***,^###^	> 0.001

Comparison in relation to the control group: *= Significant difference (P value > 0.05), ***= Very highly significant difference (P value > 0.001). Comparison in relation to the tartrazine group: #= significant difference (P value > 0.05), ###= very highly significant difference (P value > 0.001).

There was also a highly significant increase (P < 0.001) in the random blood glucose level in the TZ group compared to the control group. A decrease in mean random blood glucose values was observed in the TZ/TA group and was statistically significantly different (P < 0.05) from the control group and statistically significantly different (P < 0.001) from the TZ group (Table 2; Figure 1).

### Oxidative Enzymes

Statistical analysis of mean tissue MDA levels showed a statistically significant increase (P < 0.001) in the TZ group compared to the control group. Meanwhile, a statistically significant decrease in mean MDA values was reported in the TZ/TA group, compared to both the TZ group and the control group. There was also a statistically significant (P < 0.001) decrease in the mean values of SOD, CAT, and GSH levels in the TZ group compared to the control group. An increase in mean values of CAT and GSH was observed in the TZ/TA group and was statistically significantly different from the TZ group and also from the control group. Furthermore, an increase in mean SOD values was reported in the TZ/TA group and was statistically significantly different (P < 0.05) from the TZ group and also statistically significantly different (P < 0.001) from the control group (Table 2; Figure 1).

### LM Study

### H&E-Stained Sections

**Figure 2:**
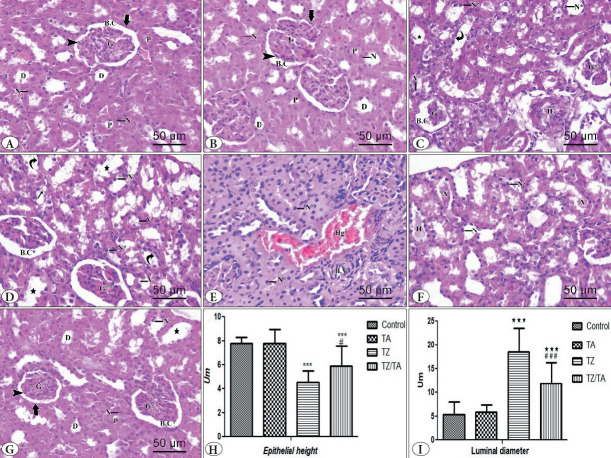
A-G: H&E-stained sections (x400, 50μm) showing the effect of TZ on the histological structure of the renal cortex and the protection provided by TA: **A)** control group; **B)** TA group; **C-F)** TZ group; **G)** TZ/TA group. Renal glomerulus (G), outer parietal layer (thick arrow), inner visceral layer (arrowhead) of Bowman’s capsule, Bowman’s space (B.C), proximal convoluted tubules (P), distal convoluted tubules (D), vesicular nuclei (N). shrunken (G*) and lobulated(G**) glomeruli, widening of Bowman’s space (B.C*), dilated tubules (star), nuclear pyknosis (N*), cytoplasmic vacuolations (V), apparent decrease in height of tubular lining cell (curved arrow), interstitial infiltration with inflammatory cells (IF), denuded basement membrane (thin arrow), peritubular interstitial hemorrhage (Hg), thick-walled blood vessel (B.V), hyaline acidophilic material cast (H), desquamated epithelial cells (X). H-I: Statistical analysis of the mean values: H) Tubular epithelial height; I) luminal diameter in different studied groups. *: Comparison in relation to the control group; ***: very highly significant difference (P value < 0.001). #: Comparison in relation to the TZ group; ###: very highly significant difference (P value < 0.001), #: (P value < 0.05).

H&E-stained sections from the kidneys of rats in the control and TA groups showed well-formed glomeruli within the renal cortex, surrounded by the visceral and parietal layers of Bowman’s capsule, which were separated from each other by Bowman’s space. The proximal convoluted tubules were lined by high simple cuboidal epithelium, with more eosinophilic cytoplasm and a narrow lumen. The distal convoluted tubules, on the other hand, were lined by low cuboidal epithelium, with pale eosinophilic cytoplasm and a relatively wide lumen. The cells lining the convoluted tubules contained vesicular nuclei (Figure 2).

In the TZ group, H&E-stained sections showed histological alterations within the renal cortex in the form of small, shrunken, and lobulated glomeruli with dilated Bowman’s space. Marked disorganization was noted in the convoluted tubules; the tubules were dilated with areas of denuded basement membrane, and the lining cells showed nuclear pyknosis, deep eosinophilic cytoplasm, cytoplasmic vacuoles and marked decrease in height. Some tubules showed deposition of acidic hyaline material and desquamated epithelial cells in their lumen. Interstitial and perivascular infiltration with inflammatory cells, interstitial hemorrhages, and dilated, thick-walled blood vessels were noted (Figure 2).

In the TZ/TA group, some histological improvement was observed within the renal cortex; there were well-formed, less lobulated glomeruli surrounded by the visceral and parietal layers of Bowman’s capsule, which were separated from each other by a less extensive Bowman’s space. Regarding the convoluted tubules, they mostly retained the normal histological structure, but some tubules still showed a wide lumen and nuclear pyknosis of the lining cells (Figure 2).

Statistical analysis of the mean height values of the cortical epithelial cells showed a very significant decrease in the mean height values of the epithelial cells of the TZ group compared to the control group. An increase in mean epithelial cell height values was demonstrated in the TZ/TA group AND was significantly different (P < 0.05) from the TZ group and very highly statistically significant (P < 0.001) from the control group (Figure 2). A highly statistically significant increase (P < 0.001) was found in the mean lumen diameter values of the TZ group compared to the control group. The TZ/TA group showed a decrease in mean lumen diameter values, which demonstrated a statistically significant difference from the TZ group and the control group (Table 3; Figure 2).

**Table 3:** Statistical analysis of the mean values of tubular epithelial height, luminal diameter, area % of collagen fibers and mesangial expansion, optical density of PAS staining, CASP3 and PCNA immunoreaction in the different studied groups using ANOVA and Tukey post hoc test

**Groups**	**Control (N=7)**	**TA (N=7)**	**TZ (N=7)**	**TZ + TA (N=7)**	**P**
**Parameter**	**Mean ±SD**	**Mean ±SD)**	**Mean ±SD)**	**Mean ±SD)**	
Epithelial height (um)	7.82 ±0.49	7.81 ±1.17	4.54±0.97***	5.89± 1.69***,^#^	< 0.001
Luminal diameter (um)	5.38 ±2.59	5.89 ±1.49	18.53±5.05***	11.93± 4.39***,^###^	< 0.001
Optical density of PAS	0.51 ±0.03	0.51 ±0.08	0.65±0.03***	0.57± 0.03*,^###^	< 0.001
Area % of mesangial expansion	11.03 ±2.61	10.89 ±2.45	24.04±6.02***	17.53± 1.66*,^#^	< 0.001
Area % of Mallory trichrome	9.75 ±1.13	9.58 ±0.68	17.01±0.85***	14.25± 0.61***,^###^	< 0.001
Optical density of CASP3	0.35 ±0.02	0.35 ±0.01	0.41±0.04***	0.37± 0.03***,^#^	< 0.001
Optical density of PCNA	0.27 ±0.02	0.28 ±0.02	0.44±0.07***	0.33± 0.03***,^###^	< 0.001

Comparison in relation to the control group: *= Significant difference (P value > 0.05), ***= Very highly significant difference (P value > 0.001). Comparison in relation to the control group: #= significant difference (P value > 0.05), ###= very highly significant difference (P value > 0.001).

### PAS-Stained Sections

**Figure 3:**
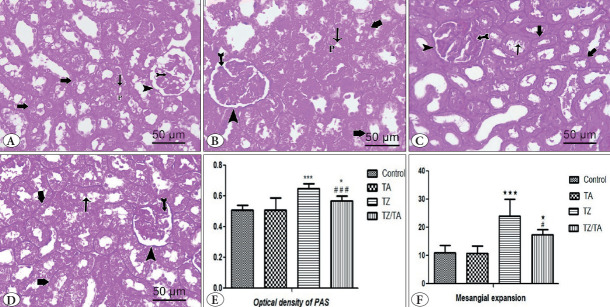
A-D: PAS-stained sections (x400, 50μm) showing the effect of TZ on glycogen deposition in the renal cortex and protection provided by TA: **A)** Control group; **B)** TA group; **C)** TZ group; **D)** TZ/TA group. **A, B)** Normal PAS staining indicating normal glycogen deposition in the glomerular mesangium (bifid arrow), outer layer of Bowman’s capsule (arrowhead) and tubular basement membrane (thick arrow). Visible brush (thin arrow) border of the proximal tubules (P) with positive PAS reaction. **C)** Strong PAS staining indicating excessive glycogen deposition in apparently thickened outer layer of Bowman’s capsules (arrowhead) and tubular basement membrane (thick arrow), disrupted brush border of the proximal tubules (thin arrow). Wide stained areas in the lobulated glomerulus indicating mesangial expansion (bifid arrow). **D)** moderate PAS staining indicating less glycogen deposition in apparently less thickened outer layer of Bowman’s capsules (arrowhead) and tubular basement membrane (thick arrow), disrupted brush border of the proximal tubules (thin arrow). Some mesangial expansion (bifid arrow). **E-F)** Statistical analysis of the mean values of E) Optical density of PAS staining and F) Area % of mesangial expansion in different studied groups. *: Comparison in relation to the control group; *: significant (P < 0.05), ***: very highly significant difference (P < 0.001). #: Comparison in relation to the TZ group; #: (P < 0.05), ###: very highly significant difference (P value < 0.001).

PAS-stained sections in the control and TA groups showed normal PAS staining of the glomerular mesangium, outer layer of Bowman’s capsule, and tubular basement membrane. A visible brush border of the proximal tubules was noted with a positive PAS reaction (Figure 3). In the TZ group, sections revealed strong staining for PAS in the apparently thickened outer layer of Bowman’s capsules, tubular basement membrane with disrupted brush border of proximal tubules. The lobulated glomeruli showed extensive stained areas indicating mesangial expansion (Figure 3). In the TZ/TA group, PAS staining was moderate in the thickened thick Bowman’s capsules and tubular basement membrane. There was still brush border disruption of the proximal tubules and some mesangial expansion of the glomeruli (Figure 3). The mean values of PAS optical density in the renal cortex showed a statistically highly significant increase (P < 0.001) in the TZ group compared to the control group. Meanwhile, a decrease in the optical density of PAS dye was observed in the TZ/TA group, which was significantly different from the TZ group and the control group (with P value < 0.001 and < 0.05, respectively) (Figure 3). Statistical analysis of the mean percentage of mesangial expansion area values in PAS-stained sections showed a statistically significant increase in the TZ group compared to the control group. However, in the TZ/TA group, there was a decrease in the mean percentage of mesangial expansion area values that was significantly different from the TZ group and the control group (Table 3; Figure 3).

### MT-Stained Sections

**Figure 4:**
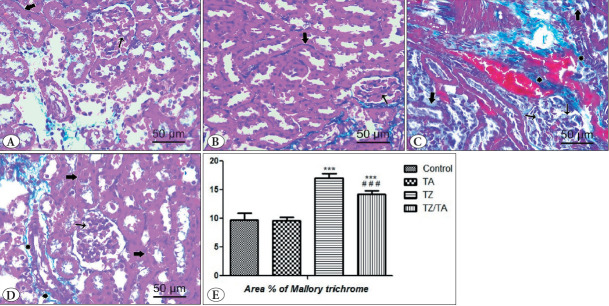
A-D) MT-stained sections (x400, 50μm) showing the effect of TZ on collagen fibers deposition in the renal cortex and protection provided by taurine: **A)** control group; **B)** TA group; **C)** TZ group; **D)** TZ/TA group. **A, B)** Minimal glomerular (thin arrow), interstitial (thick arrow) collagen fibers deposition. **C)** Excessive glomerular (thin arrow), interstitial (thick arrow) and perivascular (asterix) collagen fibers deposition. **D)** Moderate glomerular (thinarrow) and perivascular (asterix) collagen fibers deposition. Normal interstitial (thick arrow) collagen fibers **E)** Statistical analysis of the mean values of area percentage (%) of collagen fibers in different studied groups. *: Comparison in relation to the control group; ***: very highly significant difference (P < 0.001). #: Comparison in relation to the TZ group; ###: very highly significant difference (P < 0.001).

MT-stained sections from both the control and TA groups showed a normal distribution of glomerular and interstitial collagen fibers. Few blue collagen fibers were observed (Figure 4). On the other hand, in the TZ group, sections revealed glomerular fibrosis and excessive interstitial and perivascular collagen fibers stained with blue (Figure 4). In the TZ/TA group, the distribution of glomerular and perivascular collagen fibers was moderate, while a normal amount of blue-stained interstitial collagen fibers was observed (Figure 4).

The cortical area in MT-stained sections showed a statistically significant increase in the mean values of collagen fiber area percentage in the TZ group (P < 0.001) compared to the control group. However, these values were significantly decreased in the TZ/TA group compared to the TZ group and the control group (Figure 4).

#### CASP3 immunohistochemically stained sections

**Figure 5:**
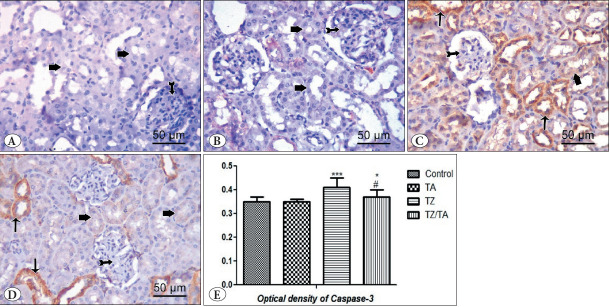
**A-D)** CASP3 immuno-stained sections (x400, 50μm) showing the effect of TZ on CASP3 immunoexpression in the renal cortex and protection provided by TA: **A)** Control group; **B)** TA group; **C)** TZ group; **D)** TZ/TA group. **A,B)** Negative cytoplasmic immunoreaction to CASP3 in the renal glomerulus (bifid arrow) and the tubular cells (thick arrow). **C)** Strong (thin arrow) to moderate (thick arrow) positive cytoplasmic immunoreaction to CASP3 in renal tubules. Negative cytoplasmic immunoreaction is observed in the renal glomerulus (bifid arrow). **D)** Weak positive cytoplasmic immunoreaction (thick arrow) to CASP3 in tubular cells is detected. Some tubules show strong positive reactions (thin arrow). Negative cytoplasmic immunoreaction is observed in the renal glomerulus (bifid arrow). E) Statistical analysis of the mean values of optical density of CASP3 in different studied groups. *: Comparison in relation to the control group; ***: Very highly significant difference (P < 0.001). #: Comparison in relation to the TZ group; ###: very highly significant difference (P < 0.001).

The control and TA groups showed negative cytoplasmic immunoreactivity for CASP3 in renal glomeruli and tubular cells (Figure 5). In the TZ group, there was a fairly strong positive cytoplasmic immunoreactivity for CASP3 in the renal tubules, while a negative cytoplasmic immunoreactivity was observed in the renal glomeruli (Figure 5). In the TZ/TA group, weak cytoplasmic immunoreactivity to CASP3 was observed in the tubular cells. Some tubules showed a strong positive response, while a negative cytoplasmic immunoreactivity was observed in the glomeruli (Figure 5).

A highly statistically significant increase in the optical density of CASP3 immunoreactivity was observed in the TZ group (P < 0.001) compared to the control group. A statistically significant decrease in the optical density of CASP3 immunoreactivity was also observed in the TZ/TA group (P < 0.05) compared to both the TZ group and the control group (Table 3; Figure 5).

#### PCNA immunohistochemically stained sections

**Figure 6:**
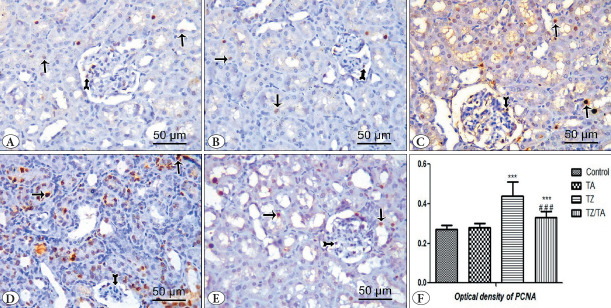
**A-E)** PCNA immuno-stained sections (x400, 50μm) showing effect of TZ on PCNA immunoexpression in the renal cortex and protection provided by TA: **A)** Control group; **B)** TA group; **C-D)** TZ group; **E)** TZ/TA group. **A,B)** Weak nuclear immunoreaction in the renal glomerulus (bifid arrow) and the tubular cells (thin arrow). **C)** Strong positive nuclear immunoreaction to PCNA in the tubular cells (thin arrow) and lobulated glomeruli (bifid arrow). **D)** Moderate positive immunoreaction in the renal glomerulus (bifid arrow) and the tubular cells (thin arrow). **F)** Statistical analysis of the mean values of optical density of PCNA in different studied groups. *: Comparison in relation to the control group; ***: very highly significant difference (P < 0.001). #: Comparison in relation to the TZ group; ###: very highly significant difference (P < 0.001).

Immunostained sections with PCNA in both control and TA groups showed weak nuclear immunoreactivity in renal glomeruli and tubular cells (Figure 6). In the TZ group, strong positive nuclear immunoreactivity for PCNA was observed in tubular cells and lobulated glomeruli, while weak nuclear immunoreactivity was observed in the shrunken glomeruli (Figure 6). In the TZ/TA group, moderate positive nuclear immunoreactivity for PCNA was observed in renal glomeruli and tubular cells (Figure 6).

Statistical analysis showed a highly statistically significant increase in the optical density of CASP3 immunoreactivity in the TZ group (P < 0.001) compared to the control group. Meanwhile, a decrease in the optical density of CASP3 immunoreactivity was observed in the TZ/TA group, which showed a statistically significant difference (P<0.001) compared with the TZ group and the control group (Table 3; Figure 6).

### EM Examination

**Figure 7:**
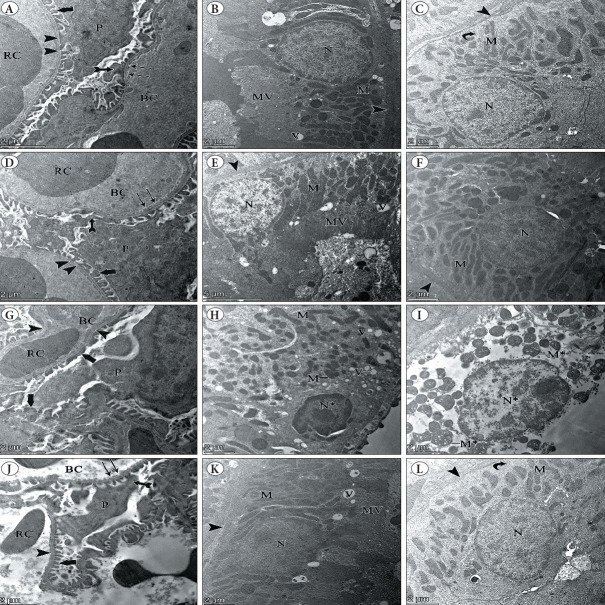
Transmission EM micrographs showing the effects of TZ on the renal cortex ultrastructure and its amelioration by TA: **A-C)** Control group; **D-F)** TA group; **G-I)** TZ group; **J-L)** TZ/TA group. **A,D)** (TEM x2000): Part of the glomerulus with its blood capillaries (BC), RBCs (RC). Fenestrated endothelial cells (thin arrow). Podocyte (P), primary foot process (bifid arrow), secondary foot processes (thick arrow), thin basement membrane (arrowhead). **B, C, E, F): B, E)** Proximal tubular epithelial cell, **C,F)** Distal tubular epithelial cell, basal round nucleus (N) with clumps of heterochromatin. Many mitochondria (M), distinct basement membrane (arrowhead), Closely packed apical microvilli (MV), prominent pinocytotic vesicles (V) basal infoldings (curved arrow). **G)** Glomerulus with its blood capillaries (BC) containing RBCs (RC). The basement membrane of glomerular capillary is thickened and lacks its fenestration (arrowhead), fusion of the secondary foot processes (thick arrow) of a podocyte (P) with the basement membrane. **H,I)** Convoluted tubular epithelial cell showing pyknotic nucleus (N*), numerous endocytotic vesicles (V). Disordered small mitochondria (M) of various shapes. swollen mitochondria (M*) with destructed cristae. **J)** Glomerulus with its blood capillary (BC) containing RBCs (RC), fenestrated endothelial cells (thin arrow), Podocyte (P), primary foot process (bifid arrow) , secondary foot processes (thick arrow), thin basement membrane (arrowhead). **K)** Proximal tubular epithelial, **L)** Distal tubular epithelial cell: Basal round nucleus (N) with clumps of heterochromatin. Many mitochondria (M), distinct basement membrane (arrowhead). Closely packed apical microvilli (MV), prominent pinocytotic vesicles (V), basal infoldings (curved arrow). **A, D, G, I, J)** (TEM x2000). B , C, F, H, K, L) (TEM x1600). E) (TEM x1250).

EM examination of ultra-thin kidney sections from the control and TA groups showed glomeruli with capillaries lined with perforated endothelial cells. Podocytes appeared with a primary process and a secondary foot process resting on a thin basement membrane. Proximal tubular epithelial cells have round, basal nuclei containing clumps of heterochromatin. Numerous rod-shaped mitochondria were found in the basal part of the cell, perpendicular to a distinct basement membrane. Closely packed microvilli and prominent pinocytotic vesicles were also seen. The distal tubular epithelial cells contained a central nucleus containing clumps of heterochromatin. Mitochondria were found in the basal portion of the cells with basal infoldings. The cells appeared on a distinct basement membrane (Figure 7).

In the TZ group, ultra-thin kidney sections showed changes in microstructure; the basement membrane of the glomerular capillaries appeared thicker and lacked its fenestration (Figure 7). The convoluted tubular epithelial cells were markedly distorted, with indistinguishable proximal and distal tubules. The cells had pyknotic nuclei with irregular borders and dense aggregates of heterochromatin. Numerous endocytic vesicles were found. Small, disordered mitochondria of various shapes were observed. Some swollen mitochondria with destructed cristae were also observed (Figure 7).

In the TZ/TA group, renal sections showed improved microarchitecture; normal glomeruli appeared with their capillaries containing red blood cells. Glomerular capillaries were lined with fenestrated endothelial cells. Podocytes appeared with primary process and secondary foot processes. The secondary foot processes appear to lie on a thin basement membrane (Figure 7). The cells of the proximal tubular epithelium had a large round nucleus with clumps of heterochromatin. Numerous rod-shaped mitochondria were found in the basal portion of the cell, perpendicular to a distinct basement membrane. Closely packed apical microvilli were also seen. However, many pinocytotic cytoplasmic vesicles were still present (Figure 7). The distal tubular epithelial cells contained a central nucleus containing clumps of heterochromatin. A few mitochondria were found in the basal portion of the cell with basal infoldings. The cell rested on a distinct basement membrane (Figure 7).

### Quantitative Real time PCR Detection of KIM-1

**Figure 8:**
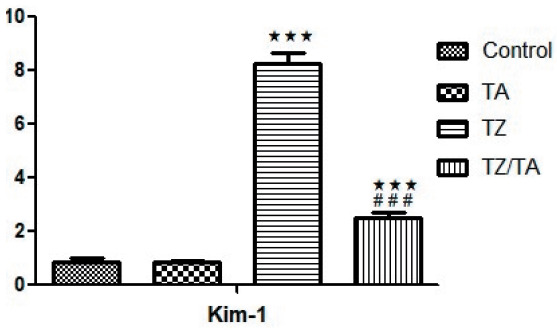
Bar chart showing the statistical analysis of the mean values of KIM-1 expression in different studied groups. *: Comparison in relation to the control group; ***: very highly significant difference (P value < 0.001). #: Comparison in relation to the TZ group; ###: very highly significant difference (P value < 0.001).

Statistical analysis of the mean KIM-1 gene expression values showed a statistically highly significant increase in the TZ group (P < 0.001) compared to the control group. A decrease in the mean KIM-1 gene expression values was recorded in the TZ/TA group, and the difference was highly significant compared to the TZ group and the control group (Figure 8).

## DISCUSSION

The present study investigated the potential protective role of TA, which is critical for many cells in almost all organs of the body, against the effects of TZ on the kidneys of adult male albino rats (14). The adult albino rat was chosen for this study because of its metabolic similarity to humans (31). Male rats were also used instead of female rats to eliminate the potential effects of hormonal changes in the female estrous cycle (32).

In this study, statistical analysis of mean random blood sugar values showed a statistically significant increase in random blood sugar levels in the TZ group compared to the control group, which is consistent with a previous study (33). It is worth noting that TZ causes hyperglycemia after two weeks of use. It also has a detrimental effect on the structure of the pancreas, impairing its endocrine functions (9). Furthermore, it stimulates glycogenolysis and gluconeogenesis (34).

TA supplementation with the TZ/TA group resulted in a significant decrease in mean random blood glucose levels compared to the TZ group. Similar results were reported by Saleh, who interpreted the hypoglycemic effect of tryptophan as resulting from increased glycogenesis, glycolysis, and glucose oxidation (35).

The levels of GSH, CAT and SOD in the present study were very highly significantly decreased in the renal tissue of the TZ group, whereas the level of MDA was very highly significantly increased in comparison to the control group. These results suggest oxidative stress state with exhaustion of the antioxidant defense. GSH, CAT, and SOD are major antioxidants and play an important role against oxidative damage to cells while MDA is a marker of oxidative stress and indicates lipid peroxidation (36). During the metabolism of TZ, sulfanilic acid and aminopyrazolone are formed, both of which have the potential to damage tissues by producing reactive oxygen species (ROS) (3). Therefore, the cells’ antioxidant defenses including GSH, SOD, and CAT begin to be consumed in an attempt to prevent cell death, while MDA levels increase as a result of lipid peroxidation caused by ROS (37). In addition, high blood sugar disrupts the electron transport chain in mitochondria, leading to increased production of ROS and induction of oxidative stress (38). Hyperglycemia with oxidative stress was found to be associated with over expression of pro-oxidative stress genes (39). Not only that, but the present study also revealed an increase in the mean values of GSH, CAT, and SOD in the TZ/TA group, which were statistically significantly different from both the TZ group and the control group. These results are consistent with other authors’ views on the role of TA in mitochondrial health (40). TA can restore the ability of mitochondrial membranes to produce ATP, which is essential for cellular metabolism.

In this study, marked disorganization was observed in the convoluted tubules of the TZ group as a result of its administration. The tubules were dilated with areas of denuded membrane. The cells lining the cortical tubules showed nuclear pyknosis, deep acidophilic cytoplasm, cytoplasmic vacuoles, and a marked decrease in height. The results were statistically confirmed, as the mean height of the epithelial cells differed significantly from the mean height of the cells in the control group. These findings represent a pattern of tubular necrosis due to single cell exfoliation, resulting in flattening of the epithelium with nuclear spacing and membrane denudation (41). Furthermore, it has been reported that the increase in the percentage of necrotic tubules in rats is directly proportional to increasing doses of TZ (42).

Cytoplasmic vacuoles in the tubular lining, reported in this work, may be considered an early sign of acute kidney injury (43). Cytoplasmic vacuolization is one of the initial responses to all types of cell damage and is caused by increased cell permeability (44). The occurrence of vacuolization is accompanied by an increase in serum urea nitrogen levels (45). Accordingly, El-Sakhawy et al., stated that the occurrence of vacuolation could represent a cellular defense against harmful compounds (17). In addition, interstitial inflammatory infiltration was observed in the TZ group in the present study. Necrosis can lead to an inflammatory reaction due to the release of cytoplasmic content from the necrotic cells, most of which are proteolytic enzymes (46). Furthermore, it has been observed that hyperglycemia and oxidative stress are associated with overexpression of pro-inflammatory genes (39). Our results also revealed interstitial hemorrhage and vascular congestion in the TZ group. Hemorrhage manifests as kidney injury caused by nephrotoxic substances, and can occur as a result of inflammation and tubular necrosis (47). The presence of dilation and congestion of blood vessels could be part of the inflammatory response to increase blood flow to the degenerated areas (48).

In this work, shrinkage of the glomeruli with widening of Bowman’s space was detected, indicating glomerular atrophy. These results are generally consistent with those reported by Himri et al. who found glomerular atrophy after oral administration of different doses of TZ (49). Salem et al. stated that mesangial cells (MCs), which are known to provide growth factors for regular cell turnover and also play a part in the production of mesangial matrix, may be the cause of glomerular atrophy (50). As a result, the harmful effects of free radicals on MCs may prevent them from performing their necessary tasks, leading to glomerular constriction, a reduction in the production of mesangial matrix, and progressive glomerular atrophy. Lobulated glomeruli were also seen in the TZ group in our study. Accentuated glomerular lobulation is a common finding in renal injury manifested by membranoproliferative glomerulonephritis and in hyperglycemic states (38). Pandir et al. stated that pathological changes that occur in renal tissue in the form of glomerular atrophy, glomerular lobulation, tubular degeneration and cellular infiltration are due to the generation of ROS and a reduction in antioxidant enzyme activities (51).

In the present study, some tubules in the TZ group showed acidophilic hyaline material deposition and desquamated epithelial cells in their lumen. These findings are in accordance with those of Elekima et al., who attributed the presence of such a hyaline material to early tubular degeneration of the nephron (52). El-Sherif and Issa, stated that the presence of hyaline material in the tubular lumen indicates the existence of lipid peroxidation and the generation of free radicals that work to break down the lipid and protein structure of intracellular membranes and hydrolyze the cytoplasm (53).

On the other hand, H&E-stained sections from the TZ/TA group in our study showed some improvement in glomerular architecture, with fewer lobulated glomeruli and less dilated Bowman’s space. Normal tubular cortical architecture was restored. Some tubules still had a slight inflammatory infiltrate, with nuclear pyknosis of the endothelial cells. These outcomes are consistent with the results of statistical analysis, which showed a statistically significant decrease in lumen diameter and an increase in epithelial height in the TZ/TA group compared to the TZ group. These findings confirm the protective role of TA for kidney tissue (12). TA can provide this protection to kidney tissue through its ability to inhibit lipid peroxidation by scavenging ROS (54). Furthermore, TA can protect kidney tissue by stabilizing the membrane and regulating osmotic pressure along with its direct antioxidant effect (55).

In the current study, strong PAS staining was observed in the apparently thickened outer layer of Bowman’s capsules and tubular basement membrane with disrupted brush border of the proximal tubules in the TZ group. Furthermore, the optical density of PAS staining was significantly different from that in the control group. Our results indicated increased renal glycogen deposition, consistent with the study by Tang et al. (56). Increased glycogen may be explained by hyperglycemia; renal glycogen deposition has been reported in cases of hyperglycemia (57). Furthermore, in the TZ group, the lobulated glomeruli showed enlarged areas stained with PAS, indicating mesangial expansion. Statistical analysis showed a significant increase in the percentage of mesangial expansion in the TZ group compared to the control group. Hyperglycemia is the primary cause of mesangial expansion, as glucose uptake stimulates matrix deposition, through upregulation of GLUT2 receptors (57). Oxidative stress also plays a critical role in the development of diabetic kidney disease and mesangial expansion (58). On the other hand, some mesangial expansion was observed in the TZ/TA group. Statistical analysis of PAS-stained sections also showed a decrease in the mean values of mesangial expansion area in the TZ/TA group, which was statistically significantly different from the TZ group. Higo et al., reported that TA reduces mesangial expansion through its antioxidant properties (59).

In this study, MT-stained sections of the control and TA groups showed a normal distribution of glomerular and interstitial collagen fibers. However, glomerular fibrosis and an increase in perivascular interstitial collagen fibers were observed in the TZ group. These results were supported by statistical analysis that showed a highly statistically significant increase in the percentage of collagen fiber area in the TZ group compared to the control group. Other authors have reported similar effects of TZ in other tissues where TZ administration has been associated with fibrotic changes (60,61). Oxidative stress and tissue damage induced by TZ administration can lead to tissue inflammation, which is followed by fibroblast activation and collagen deposition (6,62).

Coadministration of TA with TZ in the TZ/TA group animals resulted in reduced collagen fiber deposition, and MT-stained sections showed a moderate distribution of glomerular, perivascular, and interstitial collagen fibers. These results are consistent with the results of a statistical study that showed a significant decrease in the area ratio in the TZ/TA group compared to the TZ group. TA can reduce the production and release of inflammatory mediators responsible for fibrosis (63). Other authors have attributed the anti-fibrotic effect of TA to its ability to reduce oxidative stress (64).

There was a strong to moderate positive cytoplasmic immunoreactivity for CASP3 in the renal tubules, resulting in a statistically significant increase in its optical density in the TZ group compared to the control group. Other researchers have found similar results of increased CASP3 activity in the spleen tissue of rats after TZ administration (65). On the other hand, immunohistochemical staining of the TZ/TA group using CASP3 showed a weak positive cytoplasmic immunoreactivity in tubular cells. These findings are corroborated by the results of a statistical study that showed a statistically significant decrease in the optical density of CASP3 immunoreactivity in the TZ/TA group compared to the TZ group. These results are consistent with other findings from brain tissue, which indicated that TA could reduce CASP3 expression and have anti-apoptotic effects (66).

In the present study, there was strong nuclear immunoreactivity for PCNA in tubular cells and lobulated glomeruli, whereas weak nuclear immunoreactivity for PCNA was observed in shrunken glomeruli. Furthermore, there was a statistically significant increase in the optical density of PCNA immunoreactivity in the TZ group compared to the control group. These findings are in harmony with those reported by El-Sakhawy et al., who attributed this increase in proliferation to an attempt to repair damaged cells (17). Elevated PCNA immunoreactivity in the glomeruli may be due to hyperglycemia, which causes mesangial expansion. Increased PCNA immunoreactivity in the glomeruli indicates increased MCs proliferation (67).

In the present study, EM examination of ultra-thin kidney sections in the TZ group revealed ultrastructural changes; the basement membrane of glomerular capillary was apparently thickened and lacked its fenestration. The thickened basement membrane is a sign of damage caused by ROS (68). Fusion of the secondary foot processes of podocytes with basement membrane that lacked its fenestration was observed in the TZ group. These findings are in line with what was previously reported, that increased ROS production and oxidative stress cause pedicle expansion that is associated with slit pore reduction (69). Adhesions of podocytes to the basement membrane have been reported to cause segmental glomerulosclerosis through loss of the ability to separate the glomerular tuft from Bowman’s capsule (70).

The ultrastructural features of convoluted tubular epithelial cells were found to be markedly distorted with failure to differentiate between proximal and distal tubules. The cells have pyknotic nuclei with irregular borders and marginated condensed clumps of heterochromatin. There were numerous endocytic vesicles. These results are consistent with those of other authors who reported that the identification of pyknotic nuclei is a marker of toxicity (3). Excess endocytic vesicles can be explained by the fact that hyperglycemia induces glomerular hyperfiltration and hyperperfusion (71). As a result of hyperfiltration, more proteins may be present in the filtered fluid entering the tubule lumen. Consequently, the proximal tubule adapts its endocytic capacity to reabsorb the filtered protein (72).

Small, disordered mitochondria with various shapes and some swollen mitochondria with destructed cristae were observed in the TZ group. TZ inhibits mitochondrial respiration in the kidneys of mice. It also has an impact on the mitochondrial membranes’ integrity, which is necessary for sustaining essential mitochondrial functions and controlling cell death (73). The presence of some swollen mitochondria could be a result of mitochondrial fusion as a compensatory mechanism to meet the degenerated cells’ need for high metabolic activity (74).

Regarding KIM-1 protein expression, the mean values showed a significant increase in the TZ group compared to the control group. These results indicate tubular injury, based on the previous hypothesis that KIM-1 is an indicator of kidney injury. KIM-1 is a type I transmembrane protein whose expression is absent or minimal in normal conditions (75). In the present study, statistical analysis of KIM-1 expression mean values in the TZ/TA group showed a very highly significant decrease as compared to the TZ group. Similarly, other researchers found no KIM-1 in the urine after TA administration (76). Abdel-Daim et al., added that TA has the ability to guard against organ injury caused by chemical toxins through detoxification, osmoregulation, stabilization of the cell membrane, inhibition of inflammation, oxidation, and apoptosis (77).

## CONCLUSION

TZ could induce kidney injury and structural alteration mostly through induction of hyperglycemia and oxidative stress. TA can ameliorate these hazardous effects by its antioxidant and hypoglycemic properties. We recommend limiting exposure to and consumption of TZ levels in food, and suggest adding TA supplements in cases of inevitable exposure to excessive TZ levels to reduce its harmful effects on the kidneys. Further animal studies are recommended before investigating the role of TA in mitigating the kidney-damaging effects of TZ in clinical trials.

## Funding

No external funding has been received.

## Conflict of Interest

The authors declare no competing interests.

## Ethics Approval

The Institutional Animal Care and Use Committee of the Zagazig University (ZU-IACUC) approved the handling and care of animals during the experiment, approval number: ZU-IACUC/3/F/76/2021.

## References

[ref-1] Wu Limin, Xu Yufeng, Lv Xixi, Chang Xulu, Ma Xiao, Tian Xue, Shi Xi, Li Xuejun, Kong Xianghui (2021). Impacts of an azo food dye tartrazine uptake on intestinal barrier, oxidative stress, inflammatory response and intestinal microbiome in crucian carp (Carassius auratus). Ecotoxicol Environ Saf.

[ref-2] Chaudhari Sharayu S., Patil Pravin O., Bari Sanjaykumar B., Khan Zamir G. (2024). A comprehensive exploration of tartrazine detection in food products: Leveraging fluorescence nanomaterials and electrochemical sensors: Recent progress and future trends. Food Chem.

[ref-3] Khayyat Latifa, Essawy Amina, Sorour Jehan, Soffar Ahmed (2017). Tartrazine induces structural and functional aberrations and genotoxic effects in vivo. PeerJ.

[ref-4] Ameur Fatma Zohra, Mehedi Nabila, Soler Rivas Cristina, Gonzalez Antonio, Kheroua Omar, Saidi Djamel (2020). Effect of tartrazine on digestive enzymatic activities: in vivo and in vitro studies. Toxicol Res.

[ref-5] Villa Roberto Edoardo, Azimonti Giovanna, Bonos Eleftherios, Christensen Henrik, Durjava Mojca, Dusemund Birgit, Gehring Ronette, Glandorf Boet, Kouba Maryline, López-Alonso Marta, Marcon Francesca, Nebbia Carlo, Pechová Alena, Prieto-Maradona Miguel, Röhe Ilen, Theodoridou Katerina, Aquilina Gabriele, Bastos Maria, Bories Georges, Brantom Paul, Gropp Jurgen, Svensson Kettil, Tosti Luca, Finizio Antonio, Dioni Anna, Dulak-Lis Maria, Galobart Jaume, Holczknecht Orsolya, Manini Paola, Navarro-Villa Alberto, Plaza Daniel Pagés, Pizzo Fabiola, Radovnikovic Anita, Vettori Maria Vittoria, Amaduzzi Angelica, EFSA Panel on Additives and Products or Substances used in Animal Feed (FEEDAP) (2024). Safety and efficacy of a feed additive consisting of tartrazine for its use in baits for freshwater fish (GIFAP). EFSA J.

[ref-6] Barciela P., Perez-Vazquez A., Prieto M. A. (2023). Azo dyes in the food industry: Features, classification, toxicity, alternatives, and regulation. Food Chem Toxicol.

[ref-7] Abd El-Hakam FE, Farrag IM (2022). Tartrazine: Potential hepatorenal and cardiovascular toxicity and the possible protective effect of vitamin E in Wistar rats.

[ref-8] Erdemli Zeynep, Altinoz Eyup, Erdemli Mehmet Erman, Gul Mehmet, Bag Harika Gozukara, Gul Semir (2021). Ameliorative effects of crocin on tartrazine dye-induced pancreatic adverse effects: a biochemical and histological study. Environ Sci Pollut Res Int.

[ref-9] Rehman K, Ashraf A, Azam F, Akash MS (2019). Effect of food azo-dye tartrazine on physiological functions of pancreas and glucose homeostasis.

[ref-10] Abebe Worku, Mozaffari Mahmood S. (2011). Role of taurine in the vasculature: an overview of experimental and human studies. Am J Cardiovasc Dis.

[ref-11] Baliou Stella, Adamaki Maria, Ioannou Petros, Pappa Aglaia, Panayiotidis Mihalis I., Spandidos Demetrios A., Christodoulou Ioannis, Kyriakopoulos Anthony M., Zoumpourlis Vassilis (2021). Protective role of taurine against oxidative stress (Review). Mol Med Rep.

[ref-12] Heidari Reza, Jamshidzadeh Akram, Niknahad Hossein, Mardani Elnaz, Ommati Mohammad Mehdi, Azarpira Negar, Khodaei Forouzan, Zarei Azita, Ayarzadeh Maryam, Mousavi Somayeh, Abdoli Narges, Yeganeh Babak Shirazi, Saeedi Arastoo, Najibi Asma (2016). Effect of taurine on chronic and acute liver injury: Focus on blood and brain ammonia. Toxicol Rep.

[ref-13] Chesney Russell W., Han Xiaobin, Patters Andrea B. (2010). Taurine and the renal system. J Biomed Sci.

[ref-14] Froger Nicolas, Cadetti Lucia, Lorach Henri, Martins Joao, Bemelmans Alexis-Pierre, Dubus Elisabeth, Degardin Julie, Pain Dorothée, Forster Valérie, Chicaud Laurent, Ivkovic Ivana, Simonutti Manuel, Fouquet Stéphane, Jammoul Firas, Léveillard Thierry, Benosman Ryad, Sahel José-Alain, Picaud Serge (2012). Taurine provides neuroprotection against retinal ganglion cell degeneration. PLoS One.

[ref-15] Castelli Vanessa, Paladini Antonella, d'Angelo Michele, Allegretti Marcello, Mantelli Flavio, Brandolini Laura, Cocchiaro Pasquale, Cimini Annamaria, Varrassi Giustino (2021). Taurine and oxidative stress in retinal health and disease. CNS Neurosci Ther.

[ref-16] Isaac MR (2019). Effects of anabolic steroids on the histological structure of renal cortex of adult male albino rats and the possible protective role of taurine.

[ref-17] El-Sakhawy Mohamed A., Mohamed Dina W., Ahmed Yasmine H. (2019). Histological and immunohistochemical evaluation of the effect of tartrazine on the cerebellum, submandibular glands, and kidneys of adult male albino rats. Environ Sci Pollut Res Int.

[ref-18] Khamis Tarek, Hegazy Abdelmonem Awad, El-Fatah Samaa Salah Abd, Abdelfattah Eman Ramadan, Abdelfattah Marwa Mohamed Mahmoud, Fericean Liana Mihaela, Arisha Ahmed Hamed (2023). Hesperidin Mitigates Cyclophosphamide-Induced Testicular Dysfunction via Altering the Hypothalamic Pituitary Gonadal Axis and Testicular Steroidogenesis, Inflammation, and Apoptosis in Male Rats. Pharmaceuticals (Basel).

[ref-19] Sonagra AD, Zubair M, Motiani A (2025). Hexokinase Method.

[ref-20] Mariutti LRB, Cazarin B (2022). Lipid Peroxidation (TBARS) in Biological Samples. Basic Protocols in Foods and Nutrition.

[ref-21] Rahman Irfan, Kode Aruna, Biswas Saibal K. (2006). Assay for quantitative determination of glutathione and glutathione disulfide levels using enzymatic recycling method. Nat Protoc.

[ref-22] Heck Diane E., Shakarjian Michael, Kim Hong Duck, Laskin Jeffrey D., Vetrano Anna M. (2010). Mechanisms of oxidant generation by catalase. Ann N Y Acad Sci.

[ref-23] Schanne G, Demignot S, Policar C, Delsuc N (2024). Cellular evaluation of superoxide dismutase mimics as catalytic drugs: Challenges and opportunities.

[ref-24] Hegazy R, Hegazy A (2015). Hegazy' simplified method of tissue processing (consuming time and chemicals).

[ref-25] Suvarna KS, Layton C, Bancroft JD (2018). Bancroft's theory and practice of histological techniques E-Book.

[ref-26] Ersoy Tuğba, Ozmen Ozlem (2022). Immunohistochemical detection of caspase 3 and proliferating cell nuclear antigen in the intestines of dogs naturally infected with parvovirus. Vet Res Forum.

[ref-27] Woods A, Stirling J (2002). Electron microscopy: the preparative techniques, in theory and practice of histological techniques.

[ref-28] Banni Mohamed, Messaoudi Imed, Said Lamia, El Heni Jihen, Kerkeni Abdelhamid, Said Khaled (2010). Metallothionein gene expression in liver of rats exposed to cadmium and supplemented with zinc and selenium. Arch Environ Contam Toxicol.

[ref-29] Vaidya Vishal S., Ramirez Victoria, Ichimura Takaharu, Bobadilla Norma A., Bonventre Joseph V. (2006). Urinary kidney injury molecule-1: a sensitive quantitative biomarker for early detection of kidney tubular injury. Am J Physiol Renal Physiol.

[ref-30] Yuan Joshua S., Reed Ann, Chen Feng, Stewart C. Neal (2006). Statistical analysis of real-time PCR data. BMC Bioinformatics.

[ref-31] Gad SC (2006). Animal models in toxicology.

[ref-32] Hegazy AA, Abd Al Hameed EA, El-Wafaey DI, Khorshed OA (2020). Potential role of Moringa Oleifera in alleviating paracetamol-induced nephrotoxicity in rat.

[ref-33] El-Desoky Gaber E., Wabaidur Saikh M., AlOthman Zeid A., Habila Mohamed A. (2022). Evaluation of Nano-curcumin effects against Tartrazine-induced abnormalities in liver and kidney histology and other biochemical parameters. Food Sci Nutr.

[ref-34] Al-Shinnawy MS, Elkattan NA (2013). Assessment of the changes in some diagnostic parameters in male albino rats fed on an azo dye.

[ref-35] Saleh AAS (2012). Effects of taurine and/or ginseng and their mixture on lipid profile and some parameters indicative of myocardial status in streptozotocin-diabetic rats.

[ref-36] Yilgor Abdullah, Demir Canan (2024). Determination of oxidative stress level and some antioxidant activities in refractory epilepsy patients. Sci Rep.

[ref-37] Amin K. A., Abdel Hameid H., Abd Elsttar A. H. (2010). Effect of food azo dyes tartrazine and carmoisine on biochemical parameters related to renal, hepatic function and oxidative stress biomarkers in young male rats. Food Chem Toxicol.

[ref-38] Alsaad K. O., Herzenberg A. M. (2007). Distinguishing diabetic nephropathy from other causes of glomerulosclerosis: an update. J Clin Pathol.

[ref-39] Safi Sher Zaman, Shah Humaira, Qvist Rajes, Bindal Priyadarshni, Mansor Marzida, Yan Gracie Ong Siok, Ismail Ikram Shah Bin (2018). Beta Adrenergic Receptors Stimulation Attenuates Phosphorylation of NF-κB and IκBα in Hyperglycemic Endothelial Cells. Cell Physiol Biochem.

[ref-40] Jong Chian Ju, Sandal Priyanka, Schaffer Stephen W. (2021). The Role of Taurine in Mitochondria Health: More Than Just an Antioxidant. Molecules.

[ref-41] Bonsib SM, Zhou M, Magi-Galluzzi C (2007). Non-neoplastic Diseases of the Kidney. Genitourinary Pathology.

[ref-42] Rahayu MS, Wahyuni S, Fitriani I, Agung HB (2022). Effect of tartrazine on blood urea nitrogen, creatinine levels, and renal tubular necrosis in adult male Wistar rats (Rattus norvegicus): an experimental study.

[ref-43] Kiss Norbert, Hamar Péter (2016). Histopathological Evaluation of Contrast-Induced Acute Kidney Injury Rodent Models. Biomed Res Int.

[ref-44] Sakr S, Okdah A, El-Abd SF (2003). Gibberellin A3 induced histological and histochemical alterations in the liver of albino rats.

[ref-45] Ding Liang, Li Lei, Liu Senyan, Bao Xiaochen, Dickman Kathleen G., Sell Stewart S., Mei Changlin, Zhang Qing-Yu, Gu Jun, Ding Xinxin (2020). Proximal Tubular Vacuolization and Hypersensitivity to Drug-Induced Nephrotoxicity in Male Mice With Decreased Expression of the NADPH-Cytochrome P450 Reductase. Toxicol Sci.

[ref-46] Vermes I., Haanen C. (1994). Apoptosis and programmed cell death in health and disease. Adv Clin Chem.

[ref-47] Frazier Kendall S., Seely John Curtis, Hard Gordon C., Betton Graham, Burnett Roger, Nakatsuji Shunji, Nishikawa Akiyoshi, Durchfeld-Meyer Beate, Bube Axel (2012). Proliferative and nonproliferative lesions of the rat and mouse urinary system. Toxicol Pathol.

[ref-48] Moubarak R (2008). The effect of hypercholesterolemia on the rat parotid salivary gland (histopathological and immunohistochemical study).

[ref-49] Himri I, Bellahcen S, Souna FA, Belmekki F, Aziz M, Bnouham M, Zoheir J, Berkia ZO, Mekhfi H, Saalaoui EA (2011). A 90-day oral toxicity study of tartrazine, a synthetic food dye, in wistar rats.

[ref-50] Salem NA, Badawi MH, Hussein HH (2015). Protective role of propolis on diazinon induced nephrotoxicity in adult male albino rats.

[ref-51] Pandir D, Unal B, Bas H (2016). Lycopene Protects the Diabetic Rat Kidney Against Oxidative Stress-mediated Oxidative Damage Induced by Furan.

[ref-52] Elekima I, Nwachuku OE, Nduka N, Nwanjo HU, Ukwukwu D (2019). Biochemical and histological changes associated with azo food dye (tartrazine) in male albino rats.

[ref-53] El-Sherif NM, Issa NM (2015). Protective effect of rosemary (Rosmarinus officinalis) extract on naphthalene induced nephrotoxicity in adult male albino rat.

[ref-54] Aslanturk Ayse, Uzunhisarcikli Meltem (2020). Protective potential of curcumin or taurine on nephrotoxicity caused by bisphenol A. Environ Sci Pollut Res Int.

[ref-55] Yousef Hany N., Aboelwafa Hanaa R. (2017). The potential protective role of taurine against 5-fluorouracil-induced nephrotoxicity in adult male rats. Exp Toxicol Pathol.

[ref-56] Tang Lixia, Li Ke, Zhang Yan, Li Huifang, Li Ankang, Xu Yuancheng, Wei Bing (2020). Quercetin liposomes ameliorate streptozotocin-induced diabetic nephropathy in diabetic rats. Sci Rep.

[ref-57] Sullivan Mitchell A., Forbes Josephine M. (2019). Glucose and glycogen in the diabetic kidney: Heroes or villains?. EBioMedicine.

[ref-58] Jha Jay C., Banal Claudine, Chow Bryna S. M., Cooper Mark E., Jandeleit-Dahm Karin (2016). Diabetes and Kidney Disease: Role of Oxidative Stress. Antioxid Redox Signal.

[ref-59] Higo Satomi, Miyata Satoshi, Jiang Qing Yun, Kitazawa Riko, Kitazawa Sohei, Kasuga Masato (2008). Taurine administration after appearance of proteinuria retards progression of diabetic nephropathy in rats. Kobe J Med Sci.

[ref-60] Megahed RM, Barghash SS, Hasan RA (2022). Sub-chronic toxic effects of tartrazine on the heart and brain of adult male albino rats and the protective effect of vitamin.

[ref-61] Hassanin HM, Shenouda MB (2023). Histological and immunohistochemical study of tartrazine effect on the adult albino rat parotid gland and the possible protective role of omega-3 fatty acids.

[ref-62] Kalayarasan Srinivasan, Sriram Narayanan, Sudhandiran Ganapasam (2008). Diallyl sulfide attenuates bleomycin-induced pulmonary fibrosis: critical role of iNOS, NF-kappaB, TNF-alpha and IL-1beta. Life Sci.

[ref-63] Devi Shanmugam Lakshmi, Viswanathan Periyaswamy, Anuradha Carani V. (2010). Regression of liver fibrosis by taurine in rats fed alcohol: effects on collagen accumulation, selected cytokines and stellate cell activation. Eur J Pharmacol.

[ref-64] Al-Zahrani Maryam H., Balgoon Maha J., El-Sawi Nagwa M., Alshubaily Fawzia A., Jambi Ebtihaj J., Khojah Sohair M., Baljoon Raghad S., Alkhattabi Nuha A., Baz Lina A., Alharbi Asmaa A., Ahmed Amira M., Abo Elkhair Ayat M., Ismael Mohamed, Gebril Sahar M. (2023). A biochemical, theoretical and immunohistochemical study comparing the therapeutic efficacy of curcumin and taurine on T-2 toxin induced hepatotoxicity in rats. Front Mol Biosci.

[ref-65] Abd-Elhakim Yasmina M., Hashem Mohamed M., El-Metwally Abeer E., Anwar Abeer, Abo-El-Sooud Khaled, Moustafa Gihan G., Ali Haytham A. (2018). Comparative haemato-immunotoxic impacts of long-term exposure to tartrazine and chlorophyll in rats. Int Immunopharmacol.

[ref-66] Niu Xiaoli, Zheng Simin, Liu Hongtao, Li Siyuan (2018). Protective effects of taurine against inflammation, apoptosis, and oxidative stress in brain injury. Mol Med Rep.

[ref-67] Deprem T, Aksu SI, Taşçi SK, Bingöl SA, Gülmez N, Aslan Ş (2020). Immunohistochemical distributions of HGF and PCNA in the kidneys of diabetic and non-diabetic mice.

[ref-68] Youssef S, Salah M (2019). Renal Cortical structural alterations in atorvastatin-treated rats and the possible protective mechanisms of L-carnitine.

[ref-69] Ertürküner Salime Pelin, Başar Murat, Tunçdemir Matem, Seçkin İsmail (2014). The comparative effects of perindopril and catechin on mesangial matrix and podocytes in the streptozotocin induced diabetic rats. Pharmacol Rep.

[ref-70] Jefferson J. Ashley, Shankland Stuart J. (2014). The pathogenesis of focal segmental glomerulosclerosis. Adv Chronic Kidney Dis.

[ref-71] Kataoka Hiroshi, Nitta Kosaku, Hoshino Junichi (2023). Glomerular hyperfiltration and hypertrophy: an evaluation of maximum values in pathological indicators to discriminate "diseased" from "normal". Front Med (Lausanne).

[ref-72] Weisz Ora A. (2021). Endocytic adaptation to functional demand by the kidney proximal tubule. J Physiol.

[ref-73] Reyes F. G., Valim M. F., Vercesi A. E. (1996). Effect of organic synthetic food colours on mitochondrial respiration. Food Addit Contam.

[ref-74] Anan Hoda H., Zidan Rania A., Shaheen Mohammad A., Abd-El Fattah Enas A. (2016). Therapeutic efficacy of bone marrow derived mesenchymal stromal cells versus losartan on adriamycin-induced renal cortical injury in adult albino rats. Cytotherapy.

[ref-75] Bonventre Joseph V. (2008). Kidney Injury Molecule-1 (KIM-1): a specific and sensitive biomarker of kidney injury. Scand J Clin Lab Invest Suppl.

[ref-76] Latchoumycandane Calivarathan, Nagy Laura E., McIntyre Thomas M. (2014). Chronic ethanol ingestion induces oxidative kidney injury through taurine-inhibitable inflammation. Free Radic Biol Med.

[ref-77] Abdel-Daim Mohamed M., Dessouki Amina A., Abdel-Rahman Haidy G., Eltaysh Rasha, Alkahtani Saad (2019). Hepatorenal protective effects of taurine and N-acetylcysteine against fipronil-induced injuries: The antioxidant status and apoptotic markers expression in rats. Sci Total Environ.

